# Assessing brain volume changes in older women with breast cancer receiving adjuvant chemotherapy: a brain magnetic resonance imaging pilot study

**DOI:** 10.1186/s13058-018-0965-3

**Published:** 2018-05-02

**Authors:** Bihong T. Chen, Sean K. Sethi, Taihao Jin, Sunita K. Patel, Ningrong Ye, Can-Lan Sun, Russell C. Rockne, E. Mark Haacke, James C. Root, Andrew J. Saykin, Tim A. Ahles, Andrei I. Holodny, Neal Prakash, Joanne Mortimer, James Waisman, Yuan Yuan, George Somlo, Daneng Li, Richard Yang, Heidi Tan, Vani Katheria, Rachel Morrison, Arti Hurria

**Affiliations:** 1 Department of Diagnostic Radiology, City of Hope National Medical Center, Duarte, CA 91010 USA; 2 The MRI Institute for Biomedical Research, Magnetic Resonance Innovations, Inc., Detroit, MI USA; 3 Department of Population Science, City of Hope National Medical Center, Duarte, CA 91010 USA; 4 Center for Cancer and Aging, City of Hope National Medical Center, Duarte, CA 91010 USA; 5 Division of Mathematical Oncology, City of Hope National Medical Center, Duarte, CA 91010 USA; 6 Department of Biomedical Engineering, Wayne State University, Detroit, MI 48202 USA; 7 Neurocognitive Research Lab, Memorial Sloan Kettering Cancer Center, 641 Lexington Avenue, 7th Floor, New York, NY 10022 USA; 8 Center for Neuroimaging, Indiana University School of Medicine, 355 West 16th Street, Indianapolis, IN 46202 USA; 9 Department of Radiology, Memorial Sloan-Kettering Cancer Center, 641 Lexington Avenue, 7th Floor, New York, NY 10022 USA; 10 Division of Neurology, City of Hope National Medical Center, Duarte, CA 91010 USA; 11 Department of Medical Oncology, City of Hope National Medical Center, Duarte, CA 91010 USA

**Keywords:** Brain MRI, Brain volume, Chemotherapy, Cancer-related cognitive impairment, Breast cancer

## Abstract

**Background:**

Cognitive decline is among the most feared treatment-related outcomes of older adults with cancer. The majority of older patients with breast cancer self-report cognitive problems during and after chemotherapy. Prior neuroimaging research has been performed mostly in younger patients with cancer. The purpose of this study was to evaluate longitudinal changes in brain volumes and cognition in older women with breast cancer receiving adjuvant chemotherapy.

**Methods:**

Women aged ≥ 60 years with stage I–III breast cancer receiving adjuvant chemotherapy and age-matched and sex-matched healthy controls were enrolled. All participants underwent neuropsychological testing with the US National Institutes of Health (NIH) Toolbox for Cognition and brain magnetic resonance imaging (MRI) prior to chemotherapy, and again around one month after the last infusion of chemotherapy. Brain volumes were measured using Neuroreader™ software. Longitudinal changes in brain volumes and neuropsychological scores were analyzed utilizing linear mixed models.

**Results:**

A total of 16 patients with breast cancer (mean age 67.0, SD 5.39 years) and 14 age-matched and sex-matched healthy controls (mean age 67.8, SD 5.24 years) were included: 7 patients received docetaxel and cyclophosphamide (TC) and 9 received chemotherapy regimens other than TC (non-TC). There were no significant differences in segmented brain volumes between the healthy control group and the chemotherapy group pre-chemotherapy (*p* > 0.05). Exploratory hypothesis generating analyses focusing on the effect of the chemotherapy regimen demonstrated that the TC group had greater volume reduction in the temporal lobe (change = − 0.26) compared to the non-TC group (change = 0.04, *p* for interaction = 0.02) and healthy controls (change = 0.08, *p* for interaction = 0.004). Similarly, the TC group had a decrease in oral reading recognition scores (change = − 6.94) compared to the non-TC group (change = − 1.21, *p* for interaction = 0.07) and healthy controls (change = 0.09, *p* for interaction = 0.02).

**Conclusions:**

There were no significant differences in segmented brain volumes between the healthy control group and the chemotherapy group; however, exploratory analyses demonstrated a reduction in both temporal lobe volume and oral reading recognition scores among patients on the TC regimen. These results suggest that different chemotherapy regimens may have differential effects on brain volume and cognition. Future, larger studies focusing on older adults with cancer on different treatment regimens are needed to confirm these findings.

**Trial registration:**

ClinicalTrials.gov, NCT01992432. Registered on 25 November 2013. Retrospectively registered.

## Background

Cognitive decline is among the most feared symptoms in older adults undergoing treatment for cancer [1, 2]. As cancer incidence increases with age [3] and cognitive changes frequently occur following cancer systemic therapy, it is imperative to understand who is most at risk and what is the neuroanatomical basis underlying these changes. The majority of patients with breast cancer self-report cognitive problems during and after chemotherapy [4]; however, neuropsychological testing has yielded widely varying results. Different studies have reported that 13–70% of patients receiving chemotherapy demonstrate objective changes, with memory, processing speed, and executive function being the most commonly affected domains [5]. The discrepancy between patient-reported symptoms and objective results from neuropsychological testing, the wide range of results within neuropsychological testing, and the recent emphasis on individualized care all highlight the critical need to identify individuals who are especially at risk for post-therapeutic cognitive decline [6, 7]. The disparity between subjective, patient-reported cognitive problems and objective identification of cognitive problems highlights the need to better understand the neural correlates of cognitive decline.

Brain magnetic resonance imaging (MRI) can be used to identify risk factors and imaging-based biomarkers for adverse cognitive outcomes of chemotherapy treatment in patients with cancer. Adjuvant chemotherapy for breast cancer is associated with changes in structural MRI including an overall decrease of gray matter density. However, these studies have been primarily performed in younger cohorts of patients with a mean age (SD) ranging from 46.3 (6.1) to 52.9 (8.6) years [8–10]. Older adults may be at increased risk for cognitive decline. For example, a longitudinal study of individuals aged 46–86 years demonstrated that aging is associated with a reduction in brain volume, estimated at 0.5–1.5% per year in all brain structures [11] and the loss in brain volume was associated with cognitive decline [12]. However, there is a gap in knowledge regarding whether chemotherapy is associated with accelerated loss of brain volume in older adults with breast cancer.

This is a pilot longitudinal study to evaluate the association between changes in brain volume and cognition in older women with breast cancer after receiving adjuvant chemotherapy. The overall goal of the study was to evaluate the longitudinal volume measurements of brain structures that were highly associated with cognition—total gray matter, frontal lobe, and temporal lobe—among older adults with breast cancer [13]. We hypothesized that the volumes of the total gray matter, the frontal lobe, and the temporal lobe would be reduced in older women with breast cancer from pre to post-adjuvant chemotherapy and that these changes would be accompanied by decreased performance in neuropsychological testing. Recent literature shows that different chemotherapy regimens may exert different neurotoxicity profiles [14]. Thus, we performed an exploratory hypothesis-generating analysis to examine how different chemotherapy regimens affected brain volumes in our study cohort.

## Methods

The present study is a frequency matched case-control study. Cases were women aged ≥ 60 years with stage I–III breast cancer. The inclusion criteria for cases were: (1) stage I–III breast cancer in patients scheduled to receive adjuvant chemotherapy; (2) able to provide informed consent; (3) age 60 years and older; (4) of any performance status; and (5) no history of neurological or psychiatric disorders or stroke. The exclusion criteria for cases were: (1) metastatic disease or (2) MRI exclusion criteria such as claustrophobia, cardiac pacemaker, and orbital metal implants. Age-matched and sex-matched healthy controls with no history of cancer or chemotherapy exposure were recruited from the community with the same inclusion and exclusion criteria except the healthy controls did not have a cancer diagnosis. This research protocol was approved by the Institutional Review Board at City of Hope National Medical Center. Written informed consent was obtained from all study participants.

The pre-chemotherapy assessment, including a brain MRI scan and neuropsychological testing with the US National Institutes for Health (NIH) Toolbox for Cognition, was performed after surgery but before the start of adjuvant therapy (time point 1, baseline). The follow-up assessment for chemotherapy-treated patients was conducted around one month after the last infusion of chemotherapy (time point 2). The healthy control group underwent the same assessments at matched intervals.

### Brain MRI scans and brain volume measurements

#### Imaging parameters

All brain MRI scans were performed on the same 3T VERIO Siemens scanner (Siemens, Erlangen, Germany). Sagittal T1-weighted three-dimensional (3D) magnetization prepared rapid gradient echo (MPRAGE) imaging data were acquired with the following parameters: echo time (TE) = 2.94 ms, repetition time (TR) = 1900 ms, fractional anisotrophy (FA) = 9°, bandwidth = 170 Hz/pixel, imaging matrix = 256 × 176 pixels, with a voxel size of 1 × 1 × 1 mm^3^ in the axial, coronal, and sagittal planes.

#### Brain volume measurement

Brain volumes were measured using the cloud-based Neuroreader™ software (Horsens, Denmark,  https://brainreader.net/) [15–18]. This software is a commercially available and it can be used for automated volumetric measurement of segmented brain structures from 3D T1-weighted MPRAGE data. The brain volume segmentation of the imaging data was repeated three times to ensure accuracy of the automated segmentations. The output of this data analysis was carefully examined by the team for consistency. No significant inconsistency was noted during data analysis. The segmented brain structures included total gray matter, total white matter, frontal lobe, temporal lobe, parietal lobe, and occipital lobe. The volumes of bilateral lobes were combined as an overall lobe in statistical analysis.

### Neuropsychological testing

All study participants completed neuropsychological testing using the NIH Toolbox for Cognition [19]. The NIH Toolbox (http://www.healthmeasures.net/explore-measurement-systems/nih-toolbox) uses a computerized format with national standardization. The cognition battery consists of seven measures that target the subdomains of executive function, episodic memory, language, processing speed, working memory, and attention. This battery generates three composite scores and seven individual scores.

### Demographic and disease characteristics

The participants’ demographic characteristics, including age, education, race, and ethnicity, were obtained through a self-reported questionnaire. Disease stage and treatment information (the chemotherapy regimen) were obtained through medical record abstraction. The chemotherapy toxicity risk scores (as defined by the Cancer and Aging Research Group) were calculated utilizing results from the medical records and the geriatric assessment questionnaire [20, 21]. Details of the questionnaire included in this assessment have been previously published [22]. Treatment duration was calculated as days between the first infusion and last infusion of chemotherapy.

### Statistical analysis

All participants were female. The healthy controls were frequency matched to the patients with breast cancer in terms of age distribution. Unconditional logistic regression was used to compare the patients with breast cancer and healthy controls in terms of ethnicity and education. All healthy controls were white, thus the Fisher’s exact test was used to compare the race/ethnicity distribution between the patients and the healthy controls.

Statistical analysis was performed on the volume measurements from brain segmentation output using the Neuroreader™ image processing pipeline. For chemotherapy patients and healthy controls, the mean and standard deviations were presented for total white matter, total gray matter, and lobar volumes. All brain volumes were controlled using measured total intracranial volume (mTIV) and expressed as mTIV ratios. Changes were calculated as mTIV ratios at time point 2 minus mTIV ratios at time point 1. Percent changes were calculated as changes divided by mTIV ratios at time point 1. Linear mixed modeling, taking into consideration the correlation of repeated measurements within subjects, was used for longitudinal brain volume analysis [23]. Within-subject correlation was accounted for using a compound symmetry covariance structure. Time points (1 and 2) and group (patients receiving chemotherapy versus healthy controls) were both considered categorical fixed effects in the model. The interaction term of the group indicator with time point was included in the model to examine whether the changes in brain volume in the chemotherapy patient group differed significantly from those of the healthy control group. Using this linear mixed effect model with a compound symmetry covariance structure to account for correlation between repeated measurements, we examined: (1) whether there were any differences in segmented brain volumes between the chemotherapy group and the healthy control group at time point 1 and time point 2; (2) whether there were any significant changes from time point 1 to time point 2 within the chemotherapy group and the healthy control group; and (3) whether the brain volume changes differed by group (*p* for interaction). All statistical tests were two-sided. Since the main hypothesis for this study focused on total gray matter, frontal lobe, and temporal lobe, a conservative Bonferroni method was used to correct for multiple testing, with *p* values <0.01 considered statistically significant. The Bonferroni method was not applied to the statistical tests involving the neuropsychological data or any other analyses. Data were analyzed using SAS 9.3 (SAS Institute, Cary, NC, USA).

## Results

The demographic data for all study participants are summarized in Table 1. The participants consisted of 16 consecutive eligible patients with breast cancer (mean age 67, SD 5.39 years) and 14 age-matched and sex-matched healthy controls (mean age 67.8, SD 5.24 years). A total of 15 healthy controls were initially enrolled; however, one healthy control did not have the sagittal T1-weighted 3D MPRAGE sequence included in the brain MRI scan and hence was not included in the final analysis. There were no significant differences between the chemotherapy group and the healthy control group in terms of age or overall education (*p* = 0.51). All study participants were female and were right-handed. The chemotherapy group included 11 (68.8%) white women and 5 (31.2%) black women, while all healthy controls (*n* = 14) were white women (*p* = 0.04). There was no difference in ethnicity between groups. There were 5 (31.3%) patients with stage I, 8 (50.0%) patients with stage II, and 3 (18.7%) patients with stage III breast cancer. Out of the 16 patients, 7 (43.8%) received docetaxel and cyclophosphamide (TC regimen) and 9 (56.2%) received a chemotherapy regimen other than TC: 4 (25.0%) received paclitaxel and trastuzumab, and the remaining 5 patients (each 6.25%) received different chemotherapy regimens as noted in Table 1. The median duration of the chemotherapy treatment was 63 days (range 42–112 days). The median time between treatment completion and the time point 2 MRI was 22 days (range 1–42 days). The median time interval between treatment completion and neurocogitive testing was 22 days (range 1–98 days).

**Table 1:** Demographic data of the study participants

	Chemotherapy group (*n* = 16)		Healthy controls (*n* = 14)	
Variable	Number	Percent	Number	Percent
Age, years				
Mean (SD)	67.0 (5.39)	N/A	67.8 (5.24)	N/A
Range	60–82	N/A	60–78	N/A
Race				
White	11	68.8%	14	100%
Black	5	31.2%	0	0.0%
Ethnicity				
Hispanic or Latina	2	12.5%	2	14.3%
Non-Hispanic	14	87.5%	12	85.7%
Education				
High school	4	25.0%	1	7.1%
Some college or junior college	6 + 2	50.0%	4 + 4	57.1%
College degree	3	18.8%	3	21.4%
Post college	1	6.2%	2	14.4%
Stage				
I	5	31.3%	N/A	N/A
II	8	50.0%	N/A	N/A
III	3	18.7%	N/A	N/A
Regimen				
TC	7	43.75%	N/A	N/A
TCPH	1	6.25%	N/A	N/A
Paclitaxel/trastuzumab	4	25.0%	N/A	N/A
Docetaxel/cyclophosphamide/PH	1	6.25%	N/A	N/A
Carboplatin/paclitaxel	1	6.25%	N/A	N/A
ddAC – paclitaxel	1	6.25%	N/A	N/A
TAC	1	6.25%	N/A	N/A

*Abbreviations: TC* docetaxel and cyclophosphamide, *TCPH* docetaxel, carboplatin, pertuzumab, trastuzumab, *Docetaxel/cyclophosphamide/PH* docetaxel, cyclophosphamide, pertuzumab, trastuzumab, *ddAC - Paclitaxel* dose-dense doxorubicin and cyclophosphamide followed by paclitaxel, *TAC* docetaxel, doxorubicin, and cyclophosphamide, *N/A* not applicable

**Fig. 1:**
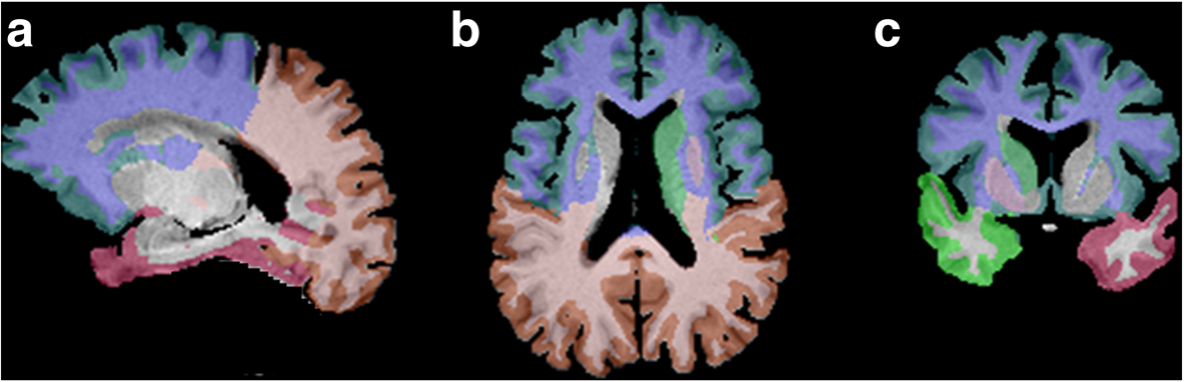
Representative images of segmented brain volumes in a study participant. This set of images shows segmented brain structures in sagittal, axial, and coronal planes (**a**, **b**, **c**)

Table 2 presents the summary of the brain volume measurements normalized by mTIV of total gray matter, total white matter, and lobar structures at time point 1 and time point 2 for both the chemotherapy group and healthy control group. The volumes of bilateral lobes were combined as an overall lobe in statistical analysis. Representative images from brain segmentation output are shown in Fig. 1. There were no significant differences between the chemotherapy group and the healthy control group (*p* > 0.05) in total gray matter, total white matter, the segmented lobar brain structures at time point 1 (baseline) or at time point 2.

**Table 2:** Measured total intracranial volume (mTIV) and brain volume measurements normalized by mTIV (mTIV ratio)

	Chemotherapy group (*n* = 16)				Healthy control group (*n* = 14)			
	Time point 1		Time point 2		Time point 1		Time point 2	
	Mean	SD	Mean	SD	Mean	SD	Mean	SD
mTIV (ml)	1718.46	114.77	1707.12	110.23	1734.76	130.92	1714.87	119.83
Volume (mTIV ratio)								
Total white matter	26.75	3.15	28.40	4.77	27.94	3.33	28.84	2.57
Total gray matter	31.70	3.39	29.64	4.21	31.24	3.81	30.26	4.62
Frontal lobe	20.23	1.86	19.89	1.66	20.17	1.52	19.90	1.70
Parietal lobe	10.70	0.79	10.63	0.81	10.78	0.81	10.70	0.90
Occipital lobe	5.29	0.51	5.30	0.59	5.48	0.48	5.46	0.53
Temporal lobe	11.05	0.71	10.96	0.72	11.30	0.82	11.38	0.82

*SD* standard deviation

Table 3 presents the longitudinal brain volume changes within each of the two groups from time point 1 to time point 2 and compares the changes between the two groups (group by time interaction). In the chemotherapy group, there were non-significant volume reductions over time in total gray matter (change = − 2.05, *p* = 0.02), significant volume reductions in the frontal lobe (change = − 0.33, *p* = 0.003), and non-significant volume increase in total white matter (change = 1.65, *p* = 0.06) from time point 1 to time point 2. However, non-significant volume reductions over time in total gray matter (change = − 0.99, *p* = 0.27) and in the frontal lobe (change = − 0.27, *p* = 0.02), and non-significant volume increase in total white matter (change = 0.90, *p* = 0.32) were also observed in the healthy control group, thus the volume changes between the two groups were not significantly different. A non-significant reduction was observed in the temporal lobe in the chemotherapy group (change = − 0.09, *p* = 0.16) and no significant reduction was observed for the healthy control group (change = 0.08, *p* = 0.25). There was a weak group-by-time interaction in the temporal lobe (*p* for interaction = 0.08). There were no significant reductions in the parietal lobe, the occipital lobe, or in total white matter in either the chemotherapy group or the healthy control group.

**Table 3:** Longitudinal volume changes (in measured total intracranial volume (mTIV) ratio) within the chemotherapy group and the healthy control group

	Chemotherapy group (*n* = 16)		Healthy control group (*n* = 14)		*p* comparing groups*
	changes in mTIV ratio (SD)	*p* value	changes in mTIV ratio (SD)	*p* value	
Total white matter	1.65 (3.63)	0.06	0.90 (2.91)	0.32	0.54
Total gray matter	−2.05 (3.38)	0.02	−0.99 (3.18)	0.27	0.38
Frontal lobe	−0.33 (0.39)	0.003	−0.27 (0.44)	0.02	0.68
Temporal lobe	−0.09 (0.31)	0.16	0.08 (0.16)	0.25	0.08
Parietal lobe	−0.07 (0.27)	0.27	−0.08 (0.22)	0.23	0.89
Occipital lobe	0.01 (0.18)	0.77	−0.02 (0.14)	0.65	0.60

*p values from comparison of volume changes between the two groups (group-by-time interaction)

**Fig. 2:**
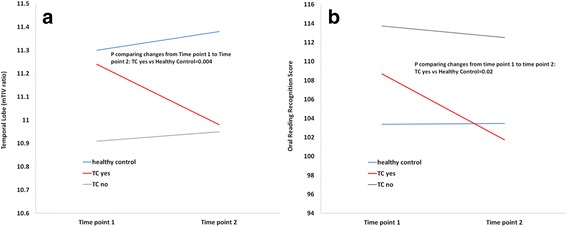
Longitudinal changes in temporal lobe volume (**a**) and Oral Reading Recognition Score (**b**). This figure shows the changes in temporal lobe volumes and scores for both the chemotherapy group including docetaxel and cyclophosphamide (TC yes) and non-TC (TC no) subgroups and the healthy control group. TC (TC yes) indicates the chemotherapy regimen consisting of docetaxel and cyclophosphamide. Non-TC (TC no) indicates a chemotherapy regimen other than the TC regimen. mTIV, measured total intracranial volume

Further exploratory analyses of the chemotherapy group revealed that the temporal lobe reduction occurred mainly among patients who received the TC regimen (docetaxel and cyclophosphamide) (change = − 0.26, *p* = 0.006) (Table 4). Compared to the healthy control group, the TC group had significant volume reduction in the temporal lobe (*p* for interaction = 0.004) (Fig. 2). The TC group had a reduction in temporal lobe volume of 2.4% from time point 1 to time point 2, while the non-TC group and healthy control group did not have a reduction. Sensitivity analysis by excluding one or two patients at a time in the TC group did not change the findings. The TC group also demonstrated significant total gray matter reduction over time (change = − 3.99, *p* = 0.002), although the reduction was not statistically significantly different from that in the non-TC group (change = − 0.56, *p* for interaction = 0.04) or the healthy control group (change = − 0.99, *p* for interaction = 0.05). There were no differences between the TC group and non-TC group in age, education, race/ethnicity, or cancer stages. There were also no significant differences between the TC group and non-TC group in the chemotherapy toxicity risk score and measures of physical function including activities of daily living measured by the Medical Outcome Study (MOS) Physical Health scale and the Instrumental Activities of Daily Living (IADL) scale. Furthermore, there were no significant differences in brain volume at baseline between the two groups. However, the patients in the TC group had a shorter chemotherapy duration (an average of 60 days), than the non-TC group (average of 86 days, *p* = 0.003).

**Table 4:** Comparison of longitudinal volume changes in the chemotherapy subgroups and the healthy control (HC) group

	Non-TC (*n* = 9)	TC (*n* = 7)	HC (*n* = 14)	TC vs. non-TC, *p*	Non-TC vs. HC, *p*	TC vs. HC, *p*
Total white matter	0.04	3.25	0.90	0.46	0.67	0.24
Total gray matter	−0.56	−3.99*	− 0.99	0.04	0.75	0.05
Frontal lobe	−0.34	−0.32	− 0.27	0.91	0.68	0.80
Temporal lobe	0.04	−0.26*	0.08	0.02	0.70	0.004
Parietal lobe	−0.13	0.01	−0.08	0.26	0.64	0.42
Occipital lobe	0.04	−0.03	−0.02	0.40	0.38	0.92

The TC subgroup (*n* = 7) included the patients on the docetaxel and cyclophosphamide chemotherapy regimen. The non-TC subgroup (*n* = 9) included the patients on a chemotherapy regimen other than the TC regimen. *p < 0.01

Table 5 summarizes all neuropsychological testing scores with the NIH Toolbox for Cognition in both the chemotherapy group and the healthy control group at time point 1 and time point 2. There were no significant differences in the neuropsychological scores between the chemotherapy group and the healthy control group at time point 1. For most of the domains, there were no significant changes over time in either the chemotherapy group or the healthy control group. The healthy control group demonstrated higher scores at time point 2 compared to time point 1, possibly due to practice effect (Table 5). However, for the chemotherapy group as a whole, most of the time point 2 scores did not increase as expected from practice effect. On the contrary, the chemotherapy group had a decrease in the oral reading recognition scores (change = − 3.71) compared to healthy controls (change = 0.09, *p* for interaction = 0.11). Further subgroup analysis showed that the reduction in oral reading recognition scores was only observed in the patients who received the TC regimen (change = − 6.94) compared to the healthy control group (change = 0.09, *p* for interaction = 0.02) (Table 6 and Fig. 2). There was no significant correlation between the volume reduction in the temporal lobe or total gray matter and decreases in the oral reading recognition score. Among patients who received chemotherapy, the Spearman’s correlation coefficient was 0.27 (*p* = 0.31); among the patients who received the TC regimen, the Spearman’s correlation coefficient was 0.17 (*p* = 0.70).

**Table 5:** Summary of neuropsychological testing data with NIH Toolbox for Cognition (score and SD)

NIH Toolbox score	Time point 1		Time point 2		Change over time		
	Chemotherapy group	Healthy controls	Chemotherapy group	Healthy controls	Chemotherapy group	Healthy controls	p**
Crystallized composite	110.87 (16.12)	107.38 (15.57)	110.56 (11.82)	107.05 (18.53)	− 0.31 (7.21)	− 0.33 (6.44)	1.00
Fluid composite	99.69 (14.43)	99.22 (10.65)	100.23 (14.62)	105.08 (15.85)	0.54 (11.66)	5.86 (11.84)	0.23
Total composite	105.10 (19.11)	101.48 (15.02)	104.14 (15.05)	105.11 (20.37)	− 0.95 (10.00)	3.63 (8.78)	0.20
Dimensional change card sort	100.66 (11.83)	101.78 (12.62)	101.70 (7.06)	106.91 (9.64)	1.04 (8.37)	5.13 (12.26)	0.29
Flanker Inhibitory control	95.78 (10.97)	96.92 (9.12)	92.50 (9.25)	99.74 (6.11)	− 3.27 (12.33)	2.81 (6.77)	0.11
Working memory	101.45 (16.31)	100.18 (16.35)	107.01 (10.64)	105.00 (17.00)	5.57 (10.19)	4.83 (14.10)	0.87
Oral reading recognition	**111.52 (11.95)**	**103.37 (12.51)**	**107.80 (12.33)**	**103.45 (14.00)**	−3.71 (5.74)*	**0.09 (6.91)**	**0.11**
Processing speed	91.29 (14.08)	96.84 (14.06)	91.14 (17.05)	95.39 (16.45)	− 0.14 (14.94)	− 1.44 (14.94)	0.81
Episodic memory	111.73 (20.06)	103.49 (15.26)	109.70 (21.33)	110.29 (25.05)	−2.02 (12.24)	6.80 (21.18)	0.17
Picture vocabulary	107.06 (14.94)	109.13 (16.63)	110.30 (8.88)	107.66 (16.52)	3.23 (9.94)	− 1.47 (6.30)	0.14

*p = 0.02

**p comparing change over time between chemotherapy group and healthy control group. Bold numbers indicate key findings

**Table 6:** Comparison of longitudinal changes in neuropsychological scores in  the chemotherapy subgroup and the healthy control (HC) group

	Non-TC (*n* = 9)	TC (*n* = 7)	HC (*n* = 14)	Non-TC vs. HC, *p*	Non-TC vs. TC, *p*	TC vs. HC, *p*
Crystallized composite	−0.17	−0.49	− 0.33	0.96	0.92	0.96
Fluid composite	−4.23	6.67	5.86	0.04	0.06	0.88
Total composite	− 2.94	1.58	3.63	0.12	0.35	0.64
Dimensional change card sort	−1.99	4.94	5.13	0.11	0.19	0.97
Flanker inhibitory control	−5.29	−0.69	2.82	0.07	0.38	0.46
Working memory	5.31	5.90	4.83	0.93	0.93	0.85
Oral reading recognition	**−1.22**	**−6.94**	**0.09**	**0.62**	**0.07**	**0.02**
Processing speed	−6.17	7.59	−1.45	0.45	0.07	0.18
Episodic memory	−4.85	1.62	6.80	0.12	0.46	0.52
Picture vocabulary	−0.05	7.46	−1.48	0.68	0.08	0.02
Picture vocabulary	−0.05	7.46	−1.48	0.68	0.08	0.02

The TC subgroup (*n* = 7) included the patients on the docetaxel and cyclophosphamide chemotherapy regimen. The non-TC subgroup (*n* = 9) included the patients on a chemotherapy regimen other than the TC regimen. Bold numbers indicate key findings

## Discussion

To the best of our knowledge, the current study is one of few prospective longitudinal studies examining changes in brain volume on brain MRI and neurocognitive function among older adults with breast cancer receiving chemotherapy. There were no significant differences in the segmented brain volumes between the healthy control group and the chemotherapy group; however, exploratory analyses demonstrated temporal lobe volume reduction in the chemotherapy subgroup of patients who received the TC regimen. Patients who received the TC regimen also had a decreased score on the oral reading recognition test.

Several of our findings are in general accord with prior structural neuroimaging studies of chemotherapy and cognition in patients with breast cancer. Most reported neuroimaging studies of cancer-related cognitive impairment were cross-sectional in design and were conducted in breast cancer survivors. These prior studies reported reduced gray matter volume [24], smaller total brain volume and gray matter volume [25], and decreased gray matter density [26]. In a cross-sectional study of breast cancer survivors treated with chemotherapy, Inagaki and colleagues showed significant differences in regional brain volume between the chemotherapy and non-chemotherapy groups after 1 year using a different imaging analysis method [27]. However, these differences in regional brain volume were not noted in a 3-year interval in the same study. A long-term survivorship study confirmed the late effects (more than 9 years) of adjuvant chemotherapy with gray matter reduction in the posterior parts of the brain in breast cancer survivors exposed to chemotherapy [24].

There are few other longitudinally designed studies of brain structural alterations in patients with breast cancer receiving adjuvant chemotherapy [9, 10, 28, 29]. McDonald and colleagues conducted a longitudinally designed study with a similar number of chemotherapy patients and healthy controls (17 patients with breast cancer on chemotherapy, 12 patients with breast cancer no chemotherapy, and 18 healthy controls), but in a younger age group at baseline: 50.6 (6.5) to 52.7 (7.2) mean years of age (SD). Their study showed acute reduction in gray matter density one month after completion of chemotherapy with a similar timeframe as our study. Their study also showed a partial recovery at 1-year follow-up assessment [9]. Additional longitudinal brain structural MRI studies presented further evidence of a similar pattern of gray matter alterations [10, 28]. Both cross-sectional and longitudinal studies have clearly identified a decrease in gray matter in the chemotherapy group compared to the non-chemotherapy cancer control group or healthy controls [13]. However, the gray matter reduction in the chemotherapy group observed in our study was not more than the reduction in the healthy control group. This lack of a significant difference could be due to our modest sample size and not having enough power to detect a modest change.

Our study showed frontal lobe volume reduction in the healthy control group as well as in the chemotherapy group. This result was not entirely surprising since our study cohort was older, ranging from 60 to 82 years of age, and older adults may experience some brain volume loss over time. Our study results were generally in line with volumetric studies of healthy aging, in which gradual gray matter atrophy has been shown as part of the normal aging process in several brain areas, especially in the frontal and temporal lobes [30, 31]. We identified frontal lobe volume loss in the healthy control group over a short interval of 2–5 months, which we had not anticipated; however, in review of the literature, a prior study showed extensive cortical reduction in the prefrontal cortex and temporal lobe after just 1 year in healthy elderly participants at 60–91 years of age, indicating accelerated brain atrophy with increasing age [32]. A study combining analyses of 56 longitudinal studies on the aging brain showed rapid brain volume loss after 60 years of age [33]. Furthermore, prior research has pointed out that some conditions, such as hypertension, subclinical depression, and preclinical neurodegenerative disease, may accelerate brain volume loss [34, 35]. These potentially confounding variables were not controlled for in the healthy control group in our study. Celle et al. reported significant blood pressure-related decreases in gray matter volume in the left superior and middle frontal gyrus [34]. There were also reports of depressive symptoms at a subclinical level in late life being associated with decreased volumes in the frontal and temporal lobes [35]. We did not have detailed blood pressure measurements or information to evaluate for any subclinical depression or preclinical neurodegenerative disease in the healthy controls in our study.

Although when examined as a whole, our study did not show a significant difference in volume changes in the temporal lobe between the chemotherapy group and the healthy control group, we did observe a significant volume reduction in the temporal lobe in patients who received the docetaxel and cyclophosphamide (TC) regimen compared to the healthy control group. Accompanying this reduction in temporal lobe volume, patients in the TC group also had reductions in oral reading recognition scores in neuropsychological testing. Other than length of the chemotherapy treatment, there were no differences between the TC and non-TC groups in terms of disease stage, age, physical functions and chemotherapy toxicity risk score. Furthermore, at baseline, there was no significant difference in temporal lobe volume between the two groups. Our study results point to a potential treatment-specific loss of temporal lobe volume and decrease in neuropsychological testing score specifically in patients treated with the TC regimen.

The temporal lobe has been shown to be one of the brain structures affected in patients with breast cancer treated with chemotherapy [27]. Brain structures in the medial temporal lobe, such as the parahippocampal gyrus, have been shown to have reduction in volume in patients treated with chemotherapy [27]. The oral reading recognition test in the NIH Toolbox for Cognition assesses reading decoding and it measures the participant’s ability to pronounce single words or letters on the computer screen [36]. The TC regimen consisted of docetaxel and cyclophosphamide and it is usually given every 21 days for four cycles. Since a taxane was also included in all of the non-TC regimens, docetaxel was less likely to be implicated. On the other hand, cyclophosphamide (which was only included in some of the non-TC regimens) is known to cross the blood-brain barrier resulting in direct neurotoxicity [37], which might have played a role in the reduction of oral reading recognition scores in the TC group. However, our explanation is mostly based on speculation and the definitive mechanisms responsible for reduction in temporal lobe volume and oral reading recognition scores in the subgroup of patients treated with the TC regimen cannot be extrapolated from this pilot study. Furthermore, we acknowledge that the oral reading recognition test is viewed as a “hold” test to estimate baseline intelligence and therefore it is possible our finding of reduced scores on this measure reflects the effects of regression to the mean rather than chemotherapy-related impact. Nevertheless, this novel finding has provided a direction for our future studies with larger cohorts to understand how different chemotherapy regimens affect brain volume and cognition in older women with breast cancer.

There were differences between our study and the published literature. For example, we did not observe a greater brain volume reduction in total gray matter and the frontal lobe in the chemotherapy group as compared to the healthy control group [13]. There are several possible reasons for the discrepancy between our data and the prior studies, including differences in study methodology (i.e. participant demographics and imaging analysis methodology), and the older age of our study participants (ranging from 60 to 82 years) than those in the reported studies. The effect of chemotherapy on brain volumes is largely unknown within a short interval (2–5 months) in this older population. Additionally, we used Neuroreader™ software for brain segmentation while the previous studies used other methods such as voxel-wise analysis [38]. In addition, Neuroreader™ reports the actual volumes of brain structures based on anatomical boundaries of specific brain structures. Therefore, it is possible that there might be significant alterations in the voxel-wise probability, which are not detected in segmented brain volumes. The heterogeneity of chemotherapy regimens for the chemotherapy group may also play a role in the varying brain volume changes, as neurotoxicity related to chemotherapy treatment may differ depending on the therapy given.

There are several limitations to this study. First of all, a modest number of participants were evaluated in this pilot study pre and post chemotherapy over a short time course of 2–5 months. Second, the majority of our participants were non-Hispanic white women, thus limiting the generalizability of our findings to other races. Third, some comorbidities such as high blood pressure and subclinical depression, which may be associated with brain volume loss in the healthy population, were not collected in this study. In addition, our study lacked a non-chemotherapy breast cancer control group, which may have helped to assess the effect of breast cancer as a source of brain structural changes. Furthermore, different methods such as voxel-based morphometry (VBM) may be utilized to assess the changes in brain volume associated with chemotherapy. It is conceivable that there might be alterations in the voxel-wise gray or white matter probability obtained with the VBM method that was not detected in our study. Last, although we did observe a larger reduction in the temporal lobe in the TC treated patients, we should caution against drawing any definitive conclusions, given the limitations of working with such a small sample size and the possibility of exaggerated effect size.

Despite these limitations, there are strengths in this study utilizing brain MRI to evaluate brain volume changes among patients receiving chemotherapy. Our study is unique in its focus on older women with breast cancer receiving different adjuvant chemotherapy regimens. Older patients with cancer are potentially vulnerable for cognitive decline, possibly from accelerated aging. However, few studies have taken advantage of utilizing the non-invasive brain MRI to study neuro-correlates of cancer-related cognitive impairment in the older population. In addition, the availability of the healthy control group in our study enabled us to compare volume changes and to identify volume reduction beyond what is expected in healthy aging.

## Conclusions

We observed no significant differences in the segmented brain volumes between patients receiving chemotherapy and the healthy control group; however, exploratory analyses demonstrated a treatment-specific reduction in both temporal lobe volume and oral reading recognition scores in the subgroup of older patients who received a regimen consisting of docetaxel and cyclophosphamide. Further studies should be conducted to examine the effect of specific chemotherapies on brain structure. Additional longitudinal studies with a larger sample size and longer follow-up intervals are needed to understand the mechanism and to validate the results from this pilot study.

## Abbreviations

ddAC - Paclitaxel: Dose-dense doxorubicin and cyclophosphamide followed by paclitaxel
Docetaxel/cyclophosphamide/PH: Docetaxel, cyclophosphamide, pertuzumab, trastuzumab
MPRAGE: Magnetization prepared rapid gradient echo
MRI: Magnetic resonance imaging
mTIV: Measured total intracranial volume
NIH: National Institutes of Health
TAC: Docetaxel, doxorubicin, and cyclophosphamide
TC: Docetaxel and cyclophosphamide
TCPH: Docetaxel, carboplatin, pertuzumab, trastuzumab
